# Danhong Injection Enhances the Therapeutic Efficacy of Mesenchymal Stem Cells in Myocardial Infarction by Promoting Angiogenesis

**DOI:** 10.3389/fphys.2018.00991

**Published:** 2018-07-26

**Authors:** Jingrui Chen, Jing Wei, Yuting Huang, Yuling Ma, Jingyu Ni, Min Li, Yan Zhu, Xiumei Gao, Guanwei Fan

**Affiliations:** ^1^First Teaching Hospital of Tianjin University of Traditional Chinese Medicine, Tianjin, China; ^2^Tianjin State Key Laboratory of Modern Chinese Medicine, Tianjin University of Traditional Chinese Medicine, Tianjin, China; ^3^Oxford Chinese Medicine Research Centre, Department of Physiology, Anatomy and Genetics, University of Oxford, Oxford, United Kingdom

**Keywords:** Danhong injection, mesenchymal stromal cells, stem cell transplantation, myocardial infarction, SDF-1/CXCR4

## Abstract

Stem cell-based therapies have the potential to dramatically transform the treatment and prognosis of myocardial infarction (MI), and mesenchymal stem cells (MSCs) have been suggested as a promising cell population to ameliorate the heart remodeling in post-MI. However, poor implantation and survival in ischemic myocardium restrict its efficacy and application. In this study, we sought to use the unique mode of action of Chinese medicine to improve this situation. Surrounding the myocardial infarct area, we performed a multi-point MSC transplantation and administered in conjunction with Danhong injection, which is mainly used for the treatment of MI. Our results showed that the MSC survival rate and cardiac function were improved significantly through the small animal imaging system and echocardiography, respectively. Moreover, histological analysis showed that MSC combined with DHI intervention significantly reduced myocardial infarct size in myocardial infarcted mice and significantly increased MSC resident. To investigate the mechanism of DHI promoting MSC survival and cell migration, PCR and WB experiments were performed. Our results showed that DHI could promote the expression of CXC chemokine receptor 4 in MSC and enhance the expression of stromal cell–derived factor-1 in myocardium, and this effect can be inhibited by AMD3100 (an SDF1/CXCR4 antagonist). Additionally, MSC in combination with DHI interfered with MI in mice and this signifies that when combined, the duo could the expression of vascular endothelial growth factor (VEGF) in the marginal zone of infarction compared with when either MSC or DHI are used individually. Based on these results, we conclude that DHI enhances the residence of MSCs in cardiac tissue by modulating the SDF1/CXCR4 signaling pathway. These findings have important therapeutic implications for Chinese medicine-assisted cell-based therapy strategies.

## Introduction

Cardiovascular disease, including atherosclerosis, stroke, and MI, is the leading cause of death in the world (Clark, 2013). In 2015, CVD accounted for one-third of all deaths, and there was an estimated 422.7 million cases of CVD and 17.92 million CVD-related deaths (Roth et al., 2017). Ischemic heart disease, especially MI, was the major cause of CVD health lost in each region across the world (Finegold et al., 2013). MI blocks the blood oxygen supply of cardiomyocytes and can kill about 25% of the cardiomyocytes in a short time, resulting in a series of serious consequences (Murry et al., 2006). After MI, fibroblasts, and endothelial cells form a dense collagenous scar to maintain wall structure, which is inflexible and non-contractile, and often lead to HF (Bergmann et al., 2009). Despite the significant progress in treatment, the prognosis of patients with MI or HF is still poor, and the current therapeutic approaches are palliative, because they do not solve the potential problems leading to loss of heart tissues (Sanganalmath and Bolli, 2013). Cardiac transplantation, a therapy being developed to eliminate the underlying cause of HF, not just to achieve damage control, is considered to be an effective treatment for the recovery of cardiac function currently. However, it is limited by insufficient donor organs and the requirement for lifelong immunosuppression (Stehlik et al., 2011; Sanganalmath and Bolli, 2013; Huang et al., 2016). Therefore, it is necessary to make efforts to develop new therapies that could repair and regenerate the myocardium.

Stem cell-based therapies have the potential to dramatically transform the treatment and prognosis of CVD, including MI or HF, via replenishing cell. Recently, the substantial preclinical and clinical studies have showed safety stem cell therapy (Khan et al., 2016). A variety of different types of stem cells with greater potential for cardiomyocyte regeneration have been tested in preclinical animal models and in humans, such as skeletal myoblasts, endothelial progenitor cells, MSC, cardiac stem/progenitor cells, embryonic stem cells (Segers and Lee, 2008; Oh et al., 2016).

Among the cell types under investigation, MSCs have been proposed as a promising cell population for cardiovascular regenerative therapy and regenerative medicinal applications (Houtgraaf et al., 2012; Squillaro et al., 2016). As a major candidate for cell therapy, MSC is not only used for heart disease, but also for the treatment of a variety of diseases characterized by fibrosis (Usunier et al., 2014; Karantalis and Hare, 2015).

Friedenstein et al. (1970), first, discovered a group of non-hematopoietic cells with multipotent and plastic-adherent stromal cells residing in the BM, commonly referred to as MSC also known as BM stromal cells. MSCs have been reported to differentiate into different types and functions of cells, including cardiomyocytes and endothelial cells (Pittenger et al., 1999; Reyes et al., 2002; Toma et al., 2002). In addition, MSC are considered to play an indispensable role in hematopoietic stem cell niches (Friedenstein et al., 1974).

In this context, the cardiogenic potential of MSCs is still controversial (Reinecke et al., 2008), and its ability to differentiate into cardiomyocytes and endothelial cells, their immunomodulatory properties, and their broad spectrum release of trophic factors have been highly thought for (Luo et al., 2017). In a large number of preclinical trials, MSC has been shown to promote angiogenesis, reduce myocardial fibrosis, and improve cardiac function in mice model with ischemic cardiomyopathy (Nagaya et al., 2005; Liu et al., 2008; Li L. et al., 2009; Mazo et al., 2010). Clinical studies have also shown that MSC therapy have significant and encouraging results for patients with ischemic cardiomyopathy, which include the improvement in physical fitness, quality of life, and ventricular remodeling (Hare et al., 2012; Li et al., 2012).

Although different kinds of stem cells have been clinically studied for cardiac repair, they all face a common challenge–donor cell loss or death (Shao et al., 2008). These problems prompted us to explore other ways to improve the efficacy of MSC in restoring cardiac structure and function, such as combination therapy (Nigro et al., 2017), especially in combination with TCM.

Danhong injection, a standard Chinese medicinal formula used in both clinical and basic research has shown promising therapeutic results in MI, including the promotion of angiogenesis, reducing infarct size, improving the micro environment of the infarct margin zone (Guan et al., 2013; Chen et al., 2016; Mao et al., 2016). Effective revascularization plays a key role in protecting cardiac function and improving long-term prognosis after MI (Jujo et al., 2008). In previous study, we found that DHI can significantly increase the expression of SDF-1 and VEGF in the myocardium of MI rats (Chen et al., 2016). Multiple stem cells are recruited to the injured area and play a repair role through SDF-1/CXFR4 signaling regulation (Askari et al., 2003; Ceradini et al., 2004). Here, we assume that DHI use the SDF1/CXCR4 signaling pathway to promote the retention and migration of exogenous MSCs, thereby improving the efficacy of MSC transplantation.

## Materials and Methods

### MSC Culture and Labeling

Human umbilical cord blood-derived MSC were purchased from Tianjin Heze Stem Cell Technology, Co., Ltd. UC-MSC cells were cultured in DMEM/F12 medium containing 10% FBS and incubated at 37°C in a 5% CO_2_ incubator for routine culture. Single cell suspension was prepared by routine digestion and centrifugation and seeded in T25 flasks at a density of 5 × 10^5^/bottle for *in vitro* and *in vivo* experiments.

In order to facilitate the transplantation of stem cell tracking, the red fluorescent dye Dil (Molecular Probes, Eugene, OR, United States) was used to label the cell membrane prior to transplantation, without affecting cell morphology, viability, and proliferation capacity, and the fluorescence signal was maintained for more than 1 month (Cheng et al., 2008; Liu et al., 2013).

### Flow Cytometric Analysis

The 5th generation UC-MSC cells were identified by flow cytometry and stained with antibodies against CD11b-PE, CD19-FITC, CD34-FITC, CD45-PE, CD73-PE, CD90-PE, and CD105-PE. Isotype-identical antibodies served as negative controls. For vitro experiments, the UC-MSC cells received a 72-h treatment with Danshensu at the concentrations of 10 μM, and the expression of CXCR4 (stained with antibodies against CXCR4-PE) was detected by flow cytometry.

### Transwell Assay

Migration of MSC toward SDF-1 was determined using Costar Transwells with 8 μm pore size (Corning, NY). The MSC cells received a pretreatment with Danshensu at the concentrations of 10 μM, then 100 μl of MSC with a total number of 20,000 cells were added into the insert. The inserts were then transferred to wells of 24-well plate containing 600 μl of 0.5% FBA-supplemented DMEM containing 100 ng/ml SDF-1. After incubation at 37°C and 5% CO_2_ for 20 h, the membranes were washed by 1× PBS, fixed by 4% paraformaldehyde (PFA) solution, and stained with DAPI. View underneath an inverted microscope and count the number of cells in different fields of view to get an average sum of cells.

### MI Model Preparation and Cell Transplantation

Male adult C57BL/6J mice (8 weeks) were purchased from Beijing Weitong Lihua Experimental Animal Technology, Co., Ltd., License number SCXK (Jing) 2012-0001. All procedures were reviewed and approved by the guidelines of Tianjin University of Traditional Chinese Medicine (TCM) Laboratory Animal Ethical Committee (TCM-LAEC20170028). MI in mice was performed, under anesthesia of 1.5–2.0% isoflurane, by LAD with mechanical ventilation as described previously (Gao et al., 2010). The procedure for sham group was totally the same but without ligating the suture. Cell transplant procedure was followed as previously reported (Wang et al., 2011). Briefly, after ligation the mice were immediately randomized to receive 15 μl of 2.0 × 10^5^ Dil-labeled UC-MSCs or saline by three injections into three areas adjacent to the infarcted tissue with a 30-gauge needle, then the chest was immediately closed and spontaneous breathing was restored.

### Animal Study Design

After the establishment of MI model, the mice were randomly divided into five groups (eight mice in each group): (1) sham group, received intraperitoneal injection of saline only; (2) model group, received intracardiac injection of saline (15 μL) and saline intraperitoneal injection; (3) MSCs group, received MSCs (2.0 × 10^5^/15 μL) intracardiac injection and saline intraperitoneal injection; (4) DHI group, received saline intracardiac injection (15 μL) and DHI intraperitoneal injection (1.5 mL/kg/day); (5) MSCs+DHI group, received MSCs (2.0 × 10^5^/15 μL) and intracardiac injection DHI intraperitoneal injection (1.5 mL/kg/day). All animals were sacrificed for histological analysis by anesthesia with 5% chloral hydrate 28 days after intraperitoneal injection.

### Echocardiographic Examination

All mice were examined by echocardiography at 2 and 4 weeks after MI under anesthesia (1.5% isoflurane in oxygen). Echocardiography was performed using a 18–38 MHz linear-array transducer with a ultra-high resolution small animal ultrasound imaging system in real time (Vevo 2100 Imaging System, VisualSonics, Toronto, ON, Canada). The standard echocardiographic acquisition procedure was reported as previously (Bauer et al., 2011).

### Hemodynamic Assessment

Mice were subjected to hemodynamics analysis by cardiac catheterization under the general anesthesia (2,2,2-Tribromoethanol) before removing the heart. Briefly, the micrometer catheter (4F, Millar Instruments) was inserted into the left ventricle through the right common carotid artery. The LV end-systolic (LVESP), end-diastolic (LVEDP) pressure and ±dp/dt_max_ were recorded by bio-function experiment system MP100-CE (BIOPAC systems, Inc., Santa Barbara, CA, United States).

### Histological and Immunofluorescent Assessments

Histological analysis was performed in paraffin-embedded sections. The heart was rinsed with PBS buffer and then perfused with 10% phosphate-buffered formalin. At the end of the perfusion, all hearts were collected and fixed in 4% PFA solution for more than 48 h, then paraffin embedded and cut into 5 μm sections. Those sections were stained with H&E and Masson for morphological analysis, and the nucleus was localized using DAPI. Fluorescence images were captured by use of the OLYMPUS DP71 inverted fluorescence microscopy.

### Quantitative Real-Time RT-PCR

The expressions of SDF-1, CXCR4, and VEGF mRNA were detected in UC-MSC and myocardium, respectively. The total RNA was extracted with TRIzol reagent (Invitrogen, United States) according to the manufacturer’s instructions, and the concentration of the total RNA was quantified with NanoDrop1000 at value ratio of 260 and 280 nm. cDNA was obtained by reverse transcription PCR using TaqMan Reverse Transcription Reagents (Roche, Switzerland) according to manufacturer’s instruction. Quantitative real-time PCR was performed using SYBR GREEN PCR Master Mix (Roche, Switzerland) and in using a CFX96^TM^ Real-Time PCR Detection System (Bio-Rad, United States). The mRNA quantity was normalized with the house-keeping gene β-actin and GAPDH. The primer sequences are listed in **Table 1**.

**Table 1:** Primers sequences used for real-time PCR.

mRNA (mice)		Sequence
SDF-1	Forward	5′ GCAGCCTTTCTCTTCTTCTGTC 3′
	Reverse	5′ ACTCCAAACTGTGCCCTTCA 3′
CXCR4	Forward	5′ CTGTCATCCCCCTGACTGAT 3′
	Reverse	5′ GAAACTGCTGGCTGAAAAGG 3′
VEGF	Forward	5′ CCTTTCCCTTTCCTCGAACT 3′
	Reverse	5′ CTCACCAAAGCCAGCACATA 3′
GAPDH	Forward	5′ AGGCCGGTGCTGAGTATGTC 3′
	Reverse	5′ TGCCTGCTTCACCACCTTCT 3′


### Western Blot

The proteins were extracted from heart samples using RIPA Lysis Buffer [Sangon Biotech (Shanghai), Co., Ltd.]. Western blotting was performed with anti-SDF-1 (ab25117, abcam), anti-CXCR4 (ab124827, abcam), and anti-GAPDH (Santa Cruz Biotechnology, Inc., Santa Cruz, CA, United States) primary antibodies and with relevant secondary anti-bodies (Santa Cruz Biotechnology, Inc.) as described previously (Chen et al., 2015).

### Small Animal Optical Imaging *in Vivo*

The fluorescence images of the transplanted Dil labeled MSC in living mice were obtained through the IVIS Lumina (PE, Waltham, MA, United States). Brief, mice transplanted with MSC underwent fluorescence imaging to detect the expression level of DIL using the IVIS at 1 week after cell transplantation under anesthesia (1.5% isoflurane in oxygen). The wavelengths of the excitation light and the wavelengths of the emitted light are 549 and 565 nm, respectively. The procedure was repeated at the second and fourth weeks, after which Dil labeled UC-MSC transplantation imaging was performed.

### Statistical Analysis

All data were presented as mean ± SD. One-way ANOVA was performed for multiple-group comparisons, and the differences in the two groups were analyzed by Student’s *t*-test using SPSS17.0 statistical software. A *p*-value less than 0.05 (*p* < 0.05) was considered as statistically significant.

## Results

### MSC Identification

**FIGURE 1:**
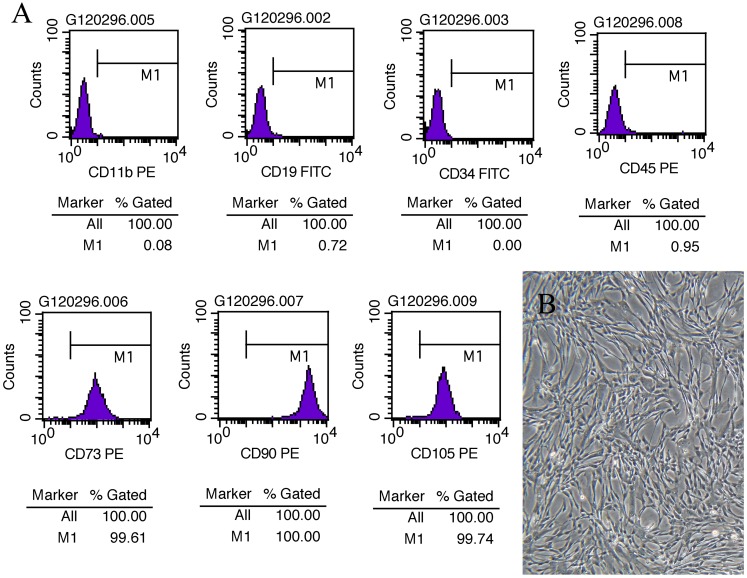
MSC assessment. **(A)** Cell surface marker of UC-MSCs at P5 showing positive for CD73, CD90, and CD105 and negative for CD19, CD11b, CD34, and CD45. **(B)** Morphological images of UC-MSCs at P5 (×100).

Microscopically, the fifth generation cells display a homogeneous spindle-shaped fibroblast like morphology (**Figure 1B**). Flow analysis results show a strong positive for cell surface marker CD73, CD90, CD105, and negative for CD 19, CD11b, CD34, CD31 (**Figure 1A**).

### MSC Plus DHI Improves Cardiac Function in Mice With Myocardial Infarction

**FIGURE 2:**
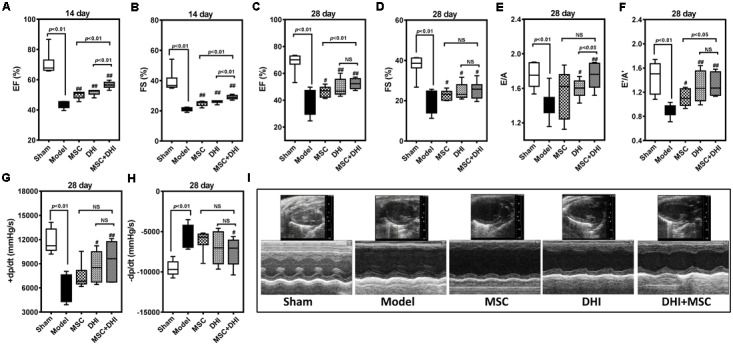
Effects of MSC combined with DHI on cardiac function and hemodynamics in MI mice. LV ejection fraction **(A)** and fractional shortening **(B)** of mice in each group after intervention for 14 days. **(C–F)** indicated LV ejection fraction (EF), fractional shortening (FS), early and late diastolic mitral flow velocity ratio (E/A), and mitral valve diastolic velocity ratio (E′/A′) in each group after 28 days of intervention, respectively. **(G,H)** The LV maximum upstroke velocity (+dp/dt_max_) and maximum descent velocity (–dp/dt_max_) were examined by cardiac catheterization. **(I)** Representative echocardiographic images (M-mode) in different groups. All values are means ± SD (*n* = 8 or *n* = 6). ^#^*p* < 0.05, ^##^*p* < 0.01 versus model group; NS means no significant difference.

To observe the effect of MSC and DHI on heart function, we detected the LV ejection fraction (EF), fractional shortening (FS), early and late diastolic mitral flow velocity ratio (E/A), the ratio of mitral valve diastolic velocity in early and late diastolic phase (E′/A′), and hemodynamic changes (pressure increase or decrease speed; ±dp/dt_max_) in mice with MI by echocardiography and LV catheterization. Indeed, compared with sham group, EF, FS, E/A, E′/A′, and +dp/dt_max_ were significantly decreased, and -dp/dt_max_ was significantly increased in the model group (*p* < 0.01). The EF, FS, E/A, and E′/A′ of mice in MSC only, DHI only, and MSC plus DHI group were improved in different degrees in 2 and 4 weeks after MI (*p* < 0.05 or *p* < 0.01), except for E/A in group B mice (**Figure 2**); compared with MSCs only or DHI only mice, the EF and FS were significantly increased in MSC plus DHI group mice in 2 weeks after MI (**Figures 2A,B**). However, compared with the only MSC group, MSC combined with DHI had no significant effect on FS, E/A, and ±dp/dt_max_ for 4 weeks of MI mice. Therefore, these data indicated that DHI promoted the role of MSC in improving cardiac function, which usually gets weaker over time in MI mice.

### Myocardium Histology

**FIGURE 3:**
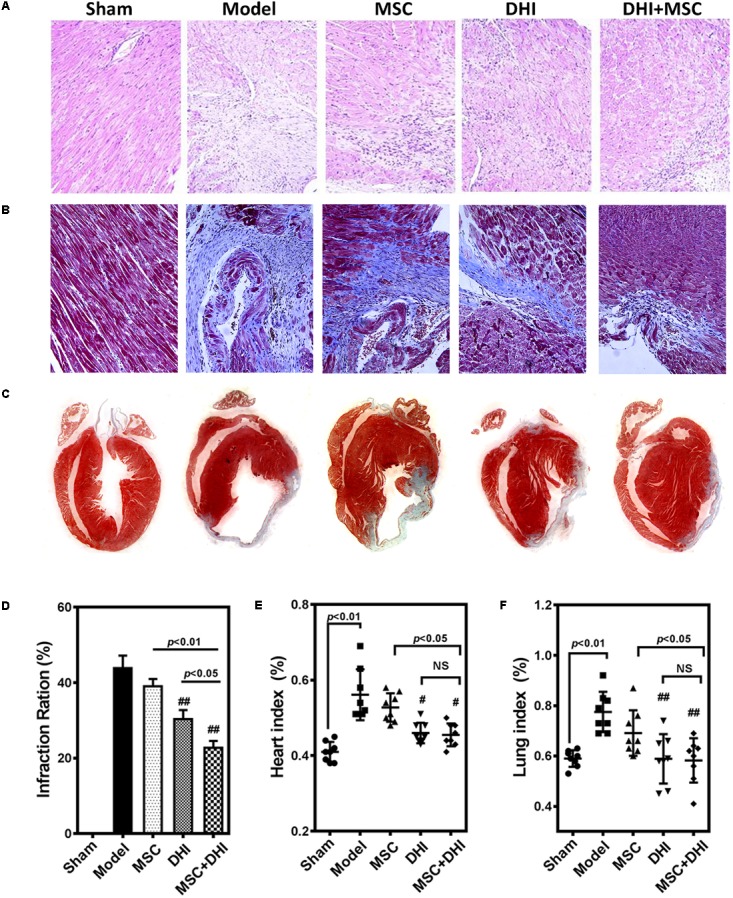
Pathological examination was performed by HE and Masson staining. **(A,B)** Representative photomicrographs of HE-stained and Masson-stained myocardium (200×). **(C)** Representative heart longitudinal panoramic view. **(D)** The infarct area ratio was quantified by midline method. **(E,F)** Representative percentage of heart and lung wet weight to body weight. All values are means ± SD (*n* = 4 or *n* = 8). ^#^*p* < 0.05, ^##^*p* < 0.01 versus model group; NS means no significant difference.

To directly assess the effect of MSC combined with DHI on the morphology of infarcted myocardium, we examined the extent of MI in mice in each group by pathological staining. As shown in HE staining results, we found that myocardial tissue structure disorder, myocardial cell edema, and fibrous fracture in mice at 4 weeks of MI, had different degrees of different degrees of improvement after intervention (**Figure 3A**). Similar to the HE staining results, compared with the model group, the infarct size of mice in DHI only and DHI plus MSC groups decreased significantly, while the infarct size of MSC only group had no significant difference. The infarct size of mice in DHI plus MSC group was significantly smaller than that in MSC only and DHI only group (**Figures 3B–D**). Moreover, the heart and lung index of mice in model group were significantly increased compared with sham group (**Figures 3E,F**). In contrast, a significantly lower heart and lung index were also observed in DHI only and MSC plus DHI group but not MSC only group. Therefore, our data suggest that transplantation of MSC combined with DHI contributes to maintaining LV geometry well, thereby reducing LV remodeling and delaying HF development.

### DHI Improves the Retention Rate of Transplanted MSC in MI Mice

**FIGURE 4:**
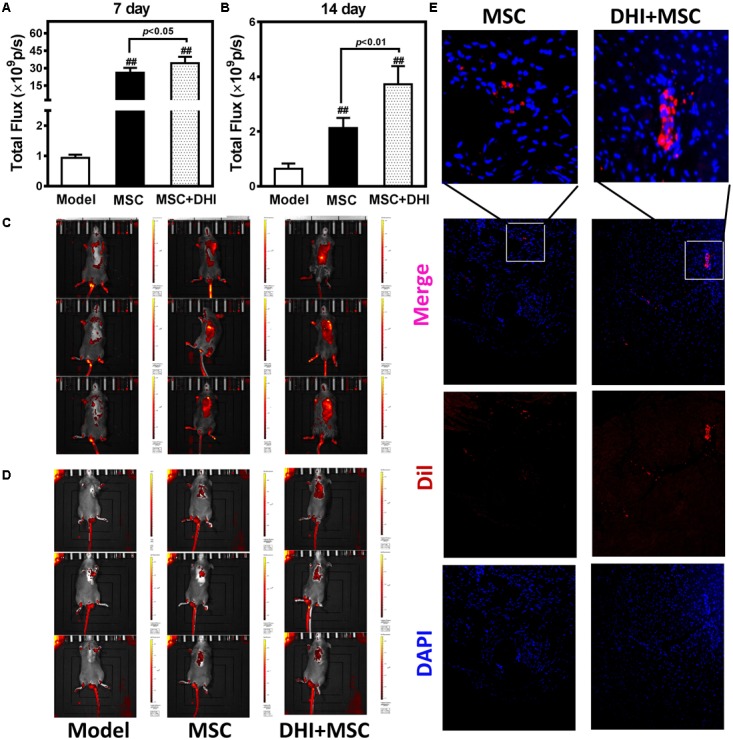
Molecular imaging of Dil-MSCs retention after transplantation. **(A,B)** Effect of DHI on MSC retention after 7 and 14 days of transplantation. **(C,D)** Representative fluorescence images of mice transplanted with MSC and MSC + DHI at 7 and 14 days were obtained by IVIS. **(E)** Representative myocardial photomicrographs of mice after transplantation of MSC and MSC + DHI for 28 days. All values are means ± SD (*n* = 8). ^##^*p* < 0.01 versus model group.

To non-invasively assess cell engraftment, the retention rate of transplanted Dil-MSCs was monitored by IVIS every week after transplantation. Compared with the sham group, Dil-MSCs transplanted along with DHI nor when applied individually could decrease the loss of Dil-MSCs significantly (*p* < 0.01). DHI significantly increased the survival rate of MSC in myocardium compared with MSC only group within 7 and 14 days after MSC transplantation (**Figures 4A–D**). Fluorescence was not detected *in vitro* after 28 days of MSC transplantation. Immunofluorescence examination showed that the number of MSC resident in the marginal area of MI in the DHI plus MSC group was significantly higher than that in the MSC only group after 28 days of transplantation (**Figure 4E**). These data indicate that the retention and survival of MSC in the myocardium is gradually reduced over time.

### DHI Promote Retention Rate of MSC via the SDF-1/CXCR4 Signaling Pathway

**FIGURE 5:**
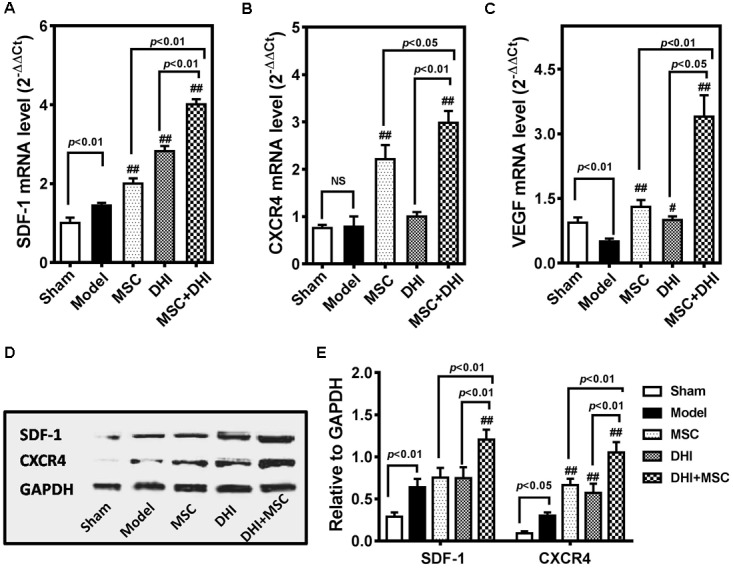
DHI promotes the survival of MSC through the SDF-1/CXCR4 signaling pathway. Quantitative real-time PCR analysis of gene expression of SDF-1 **(A)**, CXCR4 **(B)**, and VEGF **(C)** in heart samples with various treatments. **(D)** The representative western blotting bands of SDF-1 and CXCR4 protein in different groups. **(E)** Semi-quantitative analysis of SDF-1 and CXCR4 protein were normalized against GAPDH expression. All values are means ± SD (*n* = 3). ^#^*p* < 0.05, ^##^*p* < 0.01 versus model group; NS means no significant difference.

SDF-1/CXCR4 signaling is involved in the migration and survival of MSC and plays an important role in angiogenesis (Lund et al., 2014; Li et al., 2015a; Ma et al., 2015). There is also evidence that the therapeutic effect of MSCs is in part owing to their abundant secretion of SDF-1 (Tang et al., 2011; Ranganath et al., 2012; Hatzistergos et al., 2016). Therefore, we tested the hypothesis that DHI could improve the effects of MSCs on MI by regulated SDF1/CXCR4 pathway (**Figure 5**).

First, we detected the expression of SDF-1, CXCR4, and VEGF mRNA in the marginal zone of infarcted myocardium by PCR. Compared with the model group, the expression of SDF-1, CXCR4, and VEGF mRNA in each intervention group was significantly up-regulated (*p* < 0.01), except for the expression of CXCR4 mRNA in the DHI group. Moreover, the gene expression of MSC plus DHI group was significantly higher than that of MSC only group. The results of immunoblotting also showed a similar trend. As shown in **Figure 5E**, the expression of SDF-1 protein in MSC only and DHI only group did not increase significantly compared with the model group, however, that of MSC plus DHI group was significantly higher than that in in all other groups (*p* < 0.01). Nevertheless, the expression of CXCR4 protein in mice of MSC only, DHI only, and MSC plus DHI groups were significantly higher than that in model group mice (*p* < 0.01), and MSC plus DHI group was raised about 1.5-fold that of MSC only and DHI only group.

**FIGURE 6:**
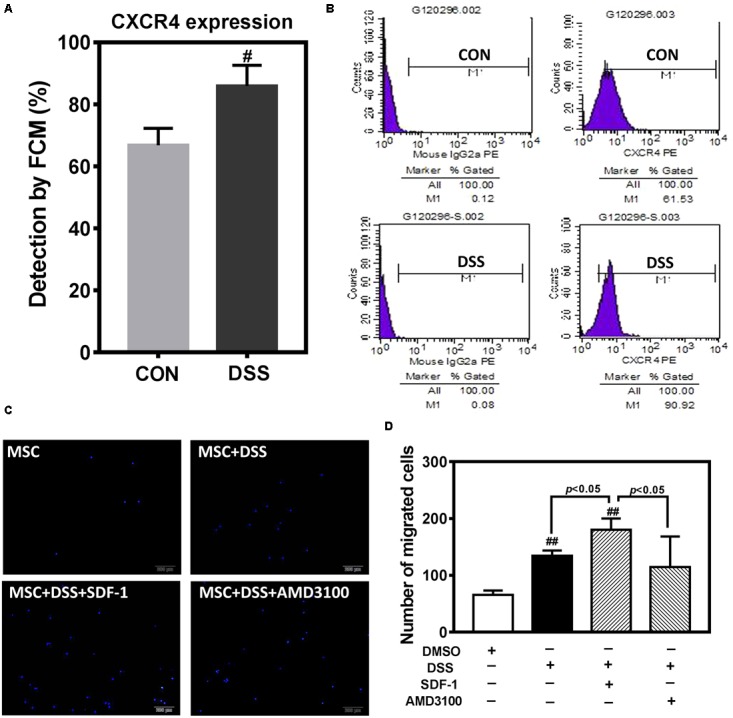
The effect of DHI main component of Danshensu on MSC cells. **(A)** Effect of Danshensu on the expression of CXCR4 protein in MSCs. **(B)** Representative flow cytometric analysis of CXCR4-PE in MSCs. **(C)** MSC cell migration was assessed by transwell assay under different interventions. **(D)** The number of MSC migration statistics. All values are means ± SD (*n* = 3). ^#^*p* < 0.05, ^##^*p* < 0.01 versus control group; NS means no significant difference.

In order to further observe the effect of DHI on MSC, we selected Danshensu (DSS), the main component of DHI (Mao et al., 2016), and incubated with MSC for 72 h. Flow cytometric analysis indicated that DSS significantly increased the expression of CXCR4 in MSC cells (**Figures 6A,B**). Next, we tested the role of DSS in MSC migration in the presence or absence of AMD3100, an inhibitor of the SDF1/CXCR4 axis (**Figure 6C**). Consistent with our conjecture, DSS can significantly increase the migration of MSC cells (*p* < 0.01). This promotion effect of MSC migration can be further enhanced by SDF-1 and was suppressed by AMD3100 (*p* < 0.05) (**Figure 6D**). These results further suggest that SDF1/CXCR4 axis plays an important role in DHI promoting MSC migration and residence.

## Discussion

In the present study, we have revealed that DHI combined with MSC intervention can significantly improve cardiac function and inhibit ventricular remodeling in murine MI models. Meanwhile, we also observed that, compared with MSC alone, MI mice receiving combination therapy were better at either MSC survival or angiogenesis. Our data clearly shows that MSC in combination with DHI can significantly improve the therapeutic effect of MSC transplantation. This benefit may be due to the regulation of the SDF-1/CXCR4 axis by DHI combined with MSC.

At present, the mechanism of action on MSC in the treatment of heart disease is mainly focused on reducing myocardial fibrosis (Molina et al., 2009), stimulating angiogenesis (Psaltis et al., 2008), and differentiating and invigorating endogenous cardiac stem cell proliferation and differentiation to restore cardiac function (Hatzistergos et al., 2010, 2016; Heldman et al., 2014; Karantalis et al., 2014). Undoubtedly, our results confirm that MSC transplantation alone can reduce myocardial fibrosis to certain extent, promote the expression of VEGF, and also have a significant improvement on cardiac function in a short period of time. However, in previous reports with other stem cells (Li et al., 2007; Li Z. et al., 2009), we could see that these effects gradually disappear with the progression of MI.

The extent of benefit of MSC transplantation is limited due to the poor retention and survival rate of MSC in myocardial tissue (Toma et al., 2002; Muller-Ehmsen et al., 2006; Quevedo et al., 2009). In addition, the route of administration is also a problem that cannot be ignored (Wang et al., 2011). Therefore, it is important to select the appropriate route of delivery and improve the survival rate of the MSC at the lesion site or to ameliorate the transplanting micro-environment for enhancing the therapeutic effect of MSCs.

At present, in order to solve the problem of poor survival rate of MSC transplantation, the combination therapy with statins (Wang et al., 2011), biomaterials (Paul et al., 2014; Yao et al., 2015), and biological effectors (Wu et al., 2011; Song et al., 2014) has been extensively explored and achieved good results. However, the joint use of TCM has not been widely explored.

The multi-effect characteristics of TCM can provide unique auxiliary effects for improving the microenvironment of MSC transplantation. We know that hypoxia and serum deprivation are unfavorable factors in the death of donor cells (Hakuno et al., 2002). We have previously reported that DHI promotes angiogenesis and improves the microenvironment in the marginal zone of MI (Chen et al., 2016). These effects of DHI may be the reason for its positive consequences on the resistance of the MSC to the hostile microenvironment and improves the survival of MSC transplants. Moreover, our results indicated that DHI administration increased the expression of VEGF, and the effects were enhanced by combination with MSC transplantation.

Stem/Progenitor cell retention and release are largely governed by the SDF-1/CXCR4 ligand–receptor interaction, which may regulate cell fate decisions (Cheng and Qin, 2012; Huang et al., 2016). Many studies show that SDF-1/CXCR4 could regulate MSC migration and homing of MSCs after MI (Marquez-Curtis and Janowska-Wieczorek, 2013; Li et al., 2015b; Zhang et al., 2016). MSCs have been widely explored as a promising treatment strategy in disorders caused by insufficient angiogenesis such as chronic wounds, stroke, and MI (Bronckaers et al., 2014). Our results show that DHI combined with MSC can significantly increase the expression of SDF1 and CXCR4 in myocardium, and the number of MSCs residing in cardiac area is significantly more than that of MSC alone transplantation. Danshensu, the main component of DHI, can promote the expression of CXCR4 in MSC cells, and its effect of promoting migration can be significantly inhibited by the selective antagonism of CXCR4 with the pharmacological agent AMD3100. These data indicate that DHI promotes the retention and migration of exogenous MSC by regulating the SDF-1/CXCR4 signaling pathway, thereby improving the efficacy of MSC transplantation.

## Conclusion

The combination of DHI with MCS transplantation could improve the heart performance in post-MI. Compared with DHI alone or MSC alone, the combined treatment strategy can achieve more benefits in regeneration and repair of MI. DHI treatment could effectively improve MSC engraftment and survival in the ischemic myocardium and also has the functional benefits from cell transplantation.

From our results, we speculate that DHI could effectively enhance the survival of the implanted MSCs in infarcted tissues, which may be related to the activation of the SDF-1/CXCR4 axis. The benefits of the combination therapy for MI may be attributed to their ability to further promote angiogenesis. The preliminary results of this study provide further evidence for the use of Chinese medicine to enhance stem cell therapy in the treatment of MI, which suggests a new strategy for stem cell therapy for MI.

## Author Contributions

GF and XG designed the experiment and were responsible for the study conception. JC and JW performed most experiments and wrote the paper. YH, YM, JN, and YZ helped to carry out experiments and analyzed the data. ML contributed to the laboratory experiments.

## Conflict of Interest Statement

The authors declare that the research was conducted in the absence of any commercial or financial relationships that could be construed as a potential conflict of interest.

## Abbreviations

BM: bone marrow
CVD: cardiovascular disease
CXCR4: CXC chemokine receptor 4
DHI: danhong injection
HF: heart failure
IVIS: *in vivo* imaging system
LAD: ligation on left anterior descending coronary artery
LV: left ventricular
MI: myocardial infarction
MSC: mesenchymal stem cells
SDF-1: stromal cell–derived factor-1
TCM: traditional Chinese medicine
VEGF: vascular endothelial growth factor.
